# Regional Disparities in Obesity Among a Heterogeneous Population of Chinese Children and Adolescents

**DOI:** 10.1001/jamanetworkopen.2021.31040

**Published:** 2021-10-26

**Authors:** Li Zhang, JingNan Chen, JianWei Zhang, Wei Wu, Ke Huang, RuiMin Chen, Mireguli Maimaiti, ShaoKe Chen, BingYan Cao, Min Zhu, ChunLin Wang, Zhe Su, Yan Liang, Hui Yao, HaiYan Wei, RongXiu Zheng, HongWei Du, FeiHong Luo, Pin Li, MinJia Mo, YunXian Yu, Ergang Wang, Robert M. Dorazio, Junfen Fu

**Affiliations:** 1 Department of Endocrinology, The Children’s Hospital, Zhejiang University School of Medicine, Hangzhou, China; 2 National Clinical Research Center for Child Health, The Children’s Hospital, Zhejiang University School of Medicine, Hangzhou, China; 3 Department of Pediatrics, Shaoxing Maternity and Child Health Care Hospital, Shaoxing, China; 4 Department of Endocrinology, Genetics and Metabolism, Fuzhou Children’s Hospital, Fujian Province, China; 5 Department of Pediatrics, the First Affiliated Hospital of Xinjiang Medical University, Ürümqi, China; 6 Department of Pediatrics, Nanning Women and Children’s Hospital, Nanning, China; 7 Department of Endocrinology, Beijing Children’s Hospital, Capital Medical University, Beijing, China; 8 Department of Endocrinology, The Children’s Hospital of Chongqing Medical University, Chongqing, China; 9 Department of Pediatrics, The First Affiliated Hospital, Zhejiang University School of Medicine, Hangzhou, China; 10 Department of Endocrinology, Shenzhen Children’s Hospital, Guangdong Province, China; 11 Department of Pediatrics, Tongji Medical College of Huazhong University of Science and Technology, Wuhan, China; 12 Department of Endocrinology, Wuhan Women and Children’s Health Care Center, Wuhan, China; 13 Department of Endocrinology, Genetics and Metabolism, Zhengzhou Children’s Hospital, Zhengzhou, China; 14 Department of Pediatrics, Tianjin Medical University General Hospital, Heping, China; 15 Department of Pediatrics, The First Bethune Hospital of Jilin University, Jilin, China; 16 Department of Endocrinology, Genetics and Metabolism, Children’s Hospital of Fudan University, Shanghai, China; 17 Department of Endocrinology, Children’s Hospital of Shanghai, Shanghai, China; 18 School of Public Health, Zhejiang University, Hangzhou, China; 19 Center for Genomics and Computational Biology, Department of Biomedical Engineering, Duke University, Durham, North Carolina

## Abstract

**Importance:**

Obesity is a public health challenge in China, but the geographical profiles of overweight and obesity among Chinese children are limited.

**Objective:**

To examine regional disparities in the prevalence of obesity among the heterogeneous population of Chinese children and adolescents to provide a more accurate profile of obesity among children in China.

**Design, Setting, and Participants:**

The Prevalence and Risk Factors for Obesity and Diabetes in Youth (PRODY) study was a cross-sectional survey study conducted from January 1, 2017, to December 31, 2019, among 201 098 children aged 3 to 18 years from 11 provinces, autonomous regions, and municipalities that produced a sample of Chinese children with a full range of ages and wide geographical coverage using a multistage, stratified, cluster-sampling design.

**Exposures:**

Five regions geographically representative of China (northern, eastern, southern, western, and central).

**Main Outcomes and Measures:**

The body weights and heights of all participants were measured. Multilevel, multinomial logistic regression models were used to estimate the prevalence of overweight and obesity.

**Results:**

Among 201 098 healthy children (105 875 boys [52.6%]; mean [SD] age, 9.8 [3.8] years) from eastern, southern, northern, central, and western China, the highest obesity prevalence was estimated for children aged 8 to 13 years in northern China (from 18.8% [95% CI, 16.2%-21.7%] to 23.6% [95% CI, 20.5%-26.9%]) and for boys aged 3 to 6 years in western China (from 18.1% [95% CI, 10.4%-29.4%] to 28.6% [95% CI, 14.3%-49.0%]). Boys had a higher prevalence than girls of obesity only in eastern and northern China, with a mean difference in prevalence of 4.6% (95% CI, 3.8%-5.4%) and 7.6% (95% CI, 6.5%-8.6%), respectively.

**Conclusions and Relevance:**

In this survey study, substantial geographic disparities in the prevalence of obesity and overweight were found among the heterogeneous population of Chinese children. The results suggest that special attention should be paid to vulnerable children and that regionally adapted interventions are needed to efficiently mitigate obesity in children.

## Key Points

**Question:**

What are the geographical characteristics of the obesity epidemic among the heterogeneous population of Chinese children and adolescents?

**Findings:**

In this cross-sectional survey that included 201 098 children aged 3 to 18 years, the highest obesity prevalence was estimated for children aged 8 to 13 years in northern China (from 18.8% to 23.6%) and for boys aged 3 to 6 years in western China (from 18.1% to 28.6%).

**Meaning:**

This study suggests that regionally adapted interventions are needed to efficiently mitigate the prevalence of obesity among the heterogeneous population of Chinese children.

## Teaser

This survey study examines regional disparities in the prevalence of obesity among the heterogeneous population of Chinese children and adolescents to provide a more accurate profile of obesity among children in China.

## Introduction

As a challenging and worldwide health problem,^1^ obesity in children and adolescents causes several medical, psychological, and social comorbidities and often leads to serious sequelae in adulthood, such as heart disease, metabolic syndrome, and steatohepatitis.^2^ Approximately 38 million children worldwide younger than 5 years of age were overweight or obese in 2019, and more than 340 million children aged 5 to 19 years were overweight or obese in 2016.^3^ The prevalence of obesity in children is increasing markedly in developing countries owing to unabated systemic and institutional factors associated with obesity.^4^ Obesity has now become a major public health challenge in China.^5^ In 1992, the national prevalence estimates of obesity and overweight among Chinese children were 3.9% for children younger than 6 years of age and 5.7% for children aged 6 to 17 years,^6^ whereas these prevalence estimates increased to 10.4% and 19%, respectively, from 2015 to 2019.^7^

To mitigate obesity among children in a large country with diverse geographic regions (such as China), it is essential to appreciate that the prevalence of overweight and obesity may differ among regions.^5^ However, evidence for the geographical profiles of overweight and obesity among Chinese children is limited. Previous findings have various limitations, including small sample sizes, incomplete regional coverage, and limited age range of participants.^8,9,10^ Hence, a study that includes children with a full range of ages and that has wide geographical coverage is crucial to assess the obesity epidemic in the heterogeneous population of Chinese children and to develop targeted strategies for the control of obesity. Using data collected in a large-scale, cross-sectional study (the Prevalence and Risk Factors for Obesity and Diabetes in Youth [PRODY] study),^11^ we aimed to evaluate the region-specific prevalence estimates of overweight and obesity that span all ages of children (3-18 years) living in western, southern, eastern, northern, and central China.

## Methods

### Study Design and Participants

As described previously,^11^ a sample of children and adolescents in China was collected in the PRODY study from January 1, 2017, to December 31, 2019, using a multistage, stratified, cluster-sampling design (eFigure 1 in the Supplement). In brief, 11 provinces, autonomous regions, and municipalities were selected during stage 1 of the study to form the sample frame. They were geographically representative of western, southern, eastern, northern, and central China and had varying levels of urbanization and economic development^12^ (eTable 1 in the Supplement). During stage 2, 34 prefectures were randomly selected from provinces or autonomous regions, followed by 35 districts, 6 county-level cities, and 10 counties randomly selected from prefectures. In addition, 7 districts were randomly selected from municipalities. Level of urbanization was not used during this stage of sampling. During stage 3, kindergartens, primary schools, and secondary schools were randomly chosen from the cities, counties, and districts selected during stage 2. All children and adolescents in the selected kindergartens or schools were invited to our study by local teachers via extensive publicity campaigns in class, and the parents or caregivers of recruited participants were contacted online by the project members. The overall response rate was 92.8% (217 127 of 234 048; 88.9% for boys [115 620 of 130 159] and 96.9% for girls [101 507 of 104 798]). The study protocol was approved by the ethics review committee of The Children’s Hospital of Zhejiang University and the cooperating institutions. Written informed consent from participants and their guardians was obtained (children younger than 5 years of age were allowed to draw symbols instead of a signature). This study followed the American Association for Public Opinion Research (AAPOR) reporting guidelines for survey studies.

### Data Collection

Demographic information (eg, name, sex, school grade, and date of birth) and medical history of participants were collected by project members through interviews. Anthropometric information (weight and height, measured by Omron HEM318; Omron Corp) was measured by physicians in our project team (W.W. and K.H.) according to standard protocols.^13^ Body mass index was calculated as weight in kilograms divided by height in meters squared. Overweight and obesity of participants were defined using age-specific body mass index cutoff values according to the Chinese criterion (eTable 2 in the Supplement).^14^ Height and weight outliers were defined as measurements that fell outside the mean ±4-fold SDs according to growth standards for Chinese children (eTable 3 in the Supplement).^15^ Underweight or normal-weight children and adolescents were assigned to a single weight-status category.

### Statistical Analysis

To summarize the characteristics of participants, we calculated percentages for categorical observations and used χ^2^ tests to compare differences among participants in different regions. Prevalence for overweight and obesity was estimated using multilevel, multinomial logistic regression models of 3 outcomes (normal weight, overweight, and obesity) and 3 levels (province, municipality, and autonomous region; prefecture and district; and county and county-level city). We estimated the prevalence for each region by including age, sex, and age-sex interactions as regressors. A 2-sided *P* = .05 was considered statistically significant. Statistical analyses were conducted using the R software program, version 4.0 (R Group for Statistical Computing).^16^

## Results

The general characteristics of our study population are presented in the Table. Our analysis included a total sample of 201 098 healthy children and adolescents (105 875 boys [52.6%] and 95 223 girls [47.4%]; mean [SD] age, 9.8 [3.8] years) from eastern, southern, northern, central, and western China. A total of 217 127 eligible participants were initially enrolled in the PRODY study between January 2017 and December 2019; however, we excluded 3220 participants who decided not to participate and 6216 participants with diagnoses of congenital metabolic disease, liver or kidney dysfunction, heart disease, or solid or hematologic tumors or who were undergoing treatment with drugs that might affect body weight (eg, orlistat, topiramate, and liraglutide). We also excluded 2121 participants with missing data on weight or height and 4472 participants with outlying height or weight values (eFigure 2 in the Supplement).

**Table.** General Characteristics and Risk Factors for the PRODY Study (2017-2019)^a^

Variable	Region of China, No. (%) of participants						*P* value
	Eastern	Northern	Central	Western	Southern	Total	
Participants	104 718 (52.1)	28 539 (14.2)	30 136 (15.0)	4950 (2.5)	32 755 (16.3)	201 098 (100)	
Status							
Normal	80 246 (76.6)	19 008 (66.6)	22 459 (74.5)	3946 (79.7)	25 948 (79.2)	151 607 (75.4)	<.001
Overweight	16 369 (15.6)	5253 (18.4)	4952 (16.4)	709 (14.3)	4408 (13.5)	31 691 (15.8)	
Obese	8103 (7.7)	4278 (15.0)	2725 (9.0)	295 (6.0)	2399 (7.3)	17 800 (8.9)	
Sex							
Male	56 390 (53.8)	14 672 (51.4)	15 594 (51.7)	2488 (50.3)	16 731 (51.1)	105 875 (52.6)	<.001
Female	48 328 (46.2)	13 867 (48.6)	14 542 (48.3)	2462 (49.7)	16 024 (48.9)	95 223 (47.4)	
Age, y							
3-7	35 962 (34.3)	13 707 (48.0)	7202 (23.9)	927 (18.7)	9199 (28.1)	66 997 (33.3)	<.001
8-13	46 868 (44.8)	11 862 (41.6)	18 867 (62.6)	2502 (50.5)	17 761 (54.2)	97 860 (48.7)	
14-18	21 888 (20.9)	2970 (10.4)	4067 (13.5)	1521 (30.7)	5795 (17.7)	36 241 (18.0)	

Abbreviation: PRODY, Prevalence and Risk Factors for Obesity and Diabetes in Youth.

^a^ The χ^2^ test was used to compare characteristic differences among participants. Underweight or normal-weight children and adolescents were assigned to a single weight-status category.

**Figure 1.**
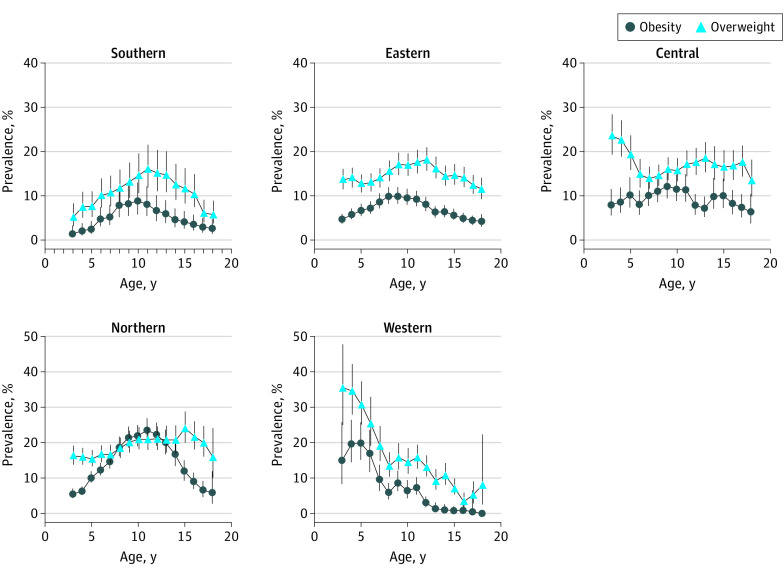
Regional Disparities in Age-Specific Prevalence Estimates of Obesity and Overweight in the Prevalence and Risk Factors for Obesity and Diabetes in Youth Study (2017-2019) The error bars indicate 95% CIs.

Our analyses revealed substantial regional disparity in obesity prevalence. The obesity prevalence for children aged 8 to 13 years in northern China was relatively high, ranging from 18.8% (95% CI, 16.2%-21.7%) to 23.6% (95% CI, 20.5%-26.9%), with 11-year-old children having the highest prevalence (Figure 1). In contrast, children from northern China aged 3 to 5 years or those older than 15 years had lower obesity prevalence estimates (3-5 years, from 5.5% [95% CI, 4.4%-7.0%] to 10.2% [95% CI, 8.7%-11.9%]; >15 years, from 5.8% [95% CI, 2.7%-11.9%] to 8.9% [95% CI, 6.9%-11.5%]). In addition, the obesity prevalence in western China was high for children aged 3 to 6 years, ranging from 15.1% (95% CI, 8.3%-25.8%) to 19.9% (95% CI, 15.1%-25.7%), and was especially high for 3- to 6-year-old boys (from 18.1% [95% CI, 10.4%-29.4%] to 28.6% [95% CI, 14.3%-49.0%]); however, children older than 6 years from western China had a lower obesity prevalence (from 0.5% [95% CI, 0.1%-3.3%] to 9.4% [95% CI, 6.4%-13.8%]) (Figure 1). The children living in southern, eastern, or central regions generally had lower obesity prevalence than those living in northern or western regions (southern region, from 1.4% [95% CI, 0.6%-3.2%] to 8.8% [95% CI, 5.8%-13.0%]; eastern region, from 4.2% [95% CI, 3.0%-5.8%] to 9.9% [95% CI, 8.2%-11.9%]; central region, from 6.3% [95% CI, 3.7%-10.4%] to 12.2% [95% CI, 9.4%-15.6%]) (Figure 1).

The regional disparity in overweight prevalence estimates was somewhat different from that of obesity. For example, children with a wide range of ages (9-17 years) living in northern China had relatively high overweight prevalence estimates, ranging from 20.1% (95% CI, 16.1%-24.7%) to 24.1% (95% CI, 20.0%-28.8%). Overweight prevalence estimates in other age groups living in northern China were also high, ranging from 15.4% (95% CI, 13.3%-17.9%) to 18.4% (95% CI, 15.7%-21.4%) (Figure 1). In contrast, in central China, the overweight prevalence estimates of young children (aged 3-5 years) were high (from 19.4% [95% CI, 15.8%-23.6%] to 23.6% [95% CI, 19.3%-28.4%]) relative to those of children older than 5 years (from 13.4% [95% CI, 9.8%-18.2%] to 19.4% [95% CI, 15.8%-23.6%]) in the region (Figure 1). Similarly, young children (aged 3-6 years) living in western China also had relatively high overweight prevalence estimates, ranging from 25.5% (95% CI, 19.1%-33.0%) to 35.6% (95% CI, 25.0%-47.8%) (Figure 1). In contrast, children aged 3 to 18 years in southern or eastern regions had relatively low overweight prevalence estimates (southern region, from 5.1% [95% CI, 3.1%-8.3%] to 16.1% [95% CI, 11.9%-21.5%]; eastern region, from 11.5% [95% CI, 9.3%-14.1%] to 18.2% [95% CI, 15.7%-21.0%]), although these prevalence estimates generally exceeded obesity prevalence estimates (Figure 1).

**Figure 2.**
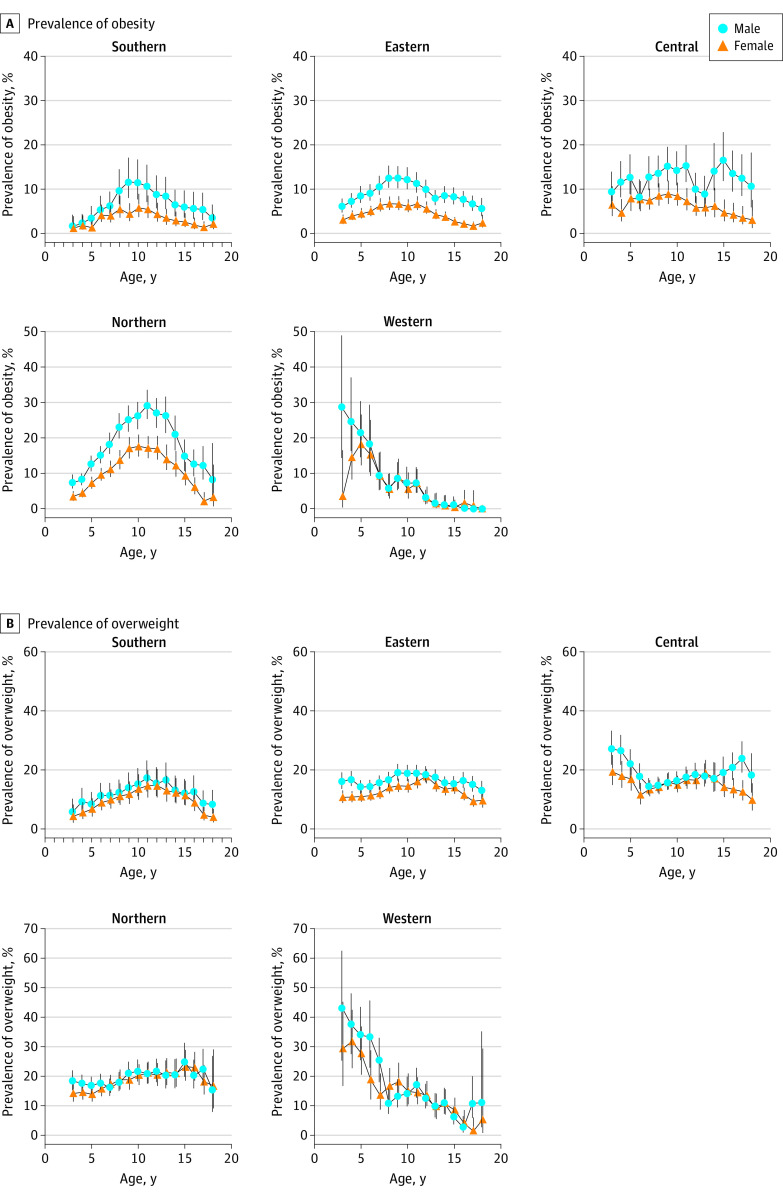
Regional Disparities in Sex- and Age-Specific Prevalence Estimates of Obesity and Overweight in the Prevalence and Risk Factors for Obesity and Diabetes in Youth Study (2017-2019) The error bars indicate 95% CIs.

Our analyses also revealed a regional disparity in the differences in obesity prevalence between boys and girls. Boys in eastern and northern China generally had higher obesity prevalence estimates than girls throughout the entire range of ages (eastern China: mean difference, 4.6% [95% CI, 3.8%-5.4%]; northern China: mean difference, 7.6% [95% CI, 6.5%-8.6%]) (Figure 2A). In contrast, in southern China, the differences in obesity prevalence between boys and girls were evident only among children aged 9 and 17 years (9 years: mean difference, 7.4% [95% CI, 5.2%-10.3%]; 17 years: mean difference, 4.0% [95% CI, 2.4%-6.4%]). In central China, these differences were evident only among children aged 4 years and 15 to 17 years (4 years: mean difference, 7.0% [95% CI, 5.4%-8.6%]; 15-17 years: mean difference, 9.9% [95% CI, 7.4%-12.9%]) (Figure 2A). In western China, differences in obesity prevalence between boys and girls were evident among young children, with boys aged 3 and 4 years having 26.0% (95% CI, 13.9%-32.4%) and 10.2% (95% CI, 8.1%-11.4%) higher prevalence estimates than girls, respectively, but not among older children. Our analyses revealed only minor differences in overweight prevalence between boys and girls in all regions, with the exception of children aged 3 to 4 years in eastern China (mean difference, 5.8% [95% CI, 5.2%-6.4%]), adolescents aged 17 years in eastern China (mean difference, 5.7% [95% CI, 5.1%-6.4%]), and adolescents aged 17 years in central China (mean difference, 11.2% [95% CI, 9.4%-13.1%]) (Figure 2B).

## Discussion

In the present study, we analyzed data collected in a large-scale nationwide survey to assess geographical differences in the prevalence of overweight and obesity among Chinese children and adolescents. The wide range of ages included in this survey allowed us to evaluate the full scope of the childhood obesity epidemic.

Our findings of regional disparities in the prevalence of overweight and obesity are generally consistent with previous nationwide surveys^8,17^ and with previous regional surveys of northern,^18,19^ eastern,^20^ southern,^21^ or northwestern China.^22^ In addition, our study corroborated previous findings that children from southern and northern China had the lowest and highest prevalence, respectively, of overweight and obesity^10^ and that children from western China had high prevalence estimates.^9^ Consistent with a recent report that demonstrated clusters of overweight and obesity in the north, northeast, and Circum-Bohai Sea regions,^8^ we provided more detailed and practical data on the geographical disparities for Chinese children. However, the prevalence estimates that we examined for central and southern China were inconsistent with a recent study,^19^ perhaps owing to the narrow age group and to a different urban-suburban composition in their study’s population.

In our study population, children aged 8 to 13 years in northern China and boys aged 3 to 6 years in western China had the highest prevalence estimates of obesity. In addition, children of all ages in northern China had relatively high overweight prevalence estimates, and young children (aged 3-5 years) from western and central China had high overweight prevalence estimates. Boys of all ages had higher obesity prevalence estimates than girls in eastern and northern China. Therefore, we believe that more health information campaigns and resources should be devoted to these vulnerable populations for the prevention and intervention of obesity. The differences in obesity prevalence estimates between boys and girls that we examined have been reported in many other studies,^17,23,24^ and these differences may be explained by stereotyped perceptions of the sexes in Chinese society.^25^ However, none of the previous studies has indicated a regional disparity in the difference in obesity prevalence estimates between boys and girls. To our knowledge, only a few studies have investigated differences of overweight and obesity prevalence estimates within age ranges, but these studies have yielded controversial conclusions.^17,19,23,26^ We speculate that the regional disparities and the limited coverage of age ranges may partly explain the diverging results. Given that socioeconomic and regional environmental characteristics (eg, per capita gross domestic product, annual mean ambient temperature, and mean altitude) contribute to the geographical disparities in risk factors for cardiovascular disease,^27^ we speculate that these characteristics may partly explain the regional differences observed in our study. Therefore, the breadth of information on the region-level socioeconomic and environmental characteristics and individual-level lifestyle, demographic, anthropometric, perinatal, and postnatal characteristics should be examined to explore the underlying reasons for the substantial regional disparities in prevalence estimates of obesity and overweight.

Prevention and intervention of childhood obesity should be public health priorities to prevent Chinese children and adolescents from experiencing multiple, obesity-related comorbid conditions. By identifying vulnerable populations of Chinese children, our findings suggest that geographically targeted intervention strategies will help to address the obesity burden in China more efficiently.

### Strengths and Limitations

Our study has some strengths, including its large sample size and broad geographical coverage, which allowed us to assess regional disparities in prevalence estimates of overweight and obesity for the heterogeneous population of Chinese children. In addition, the PRODY study survey included body weights and heights measured by physicians, resulting in more accurate estimates of body mass index than self-reported or parental-reported weights and heights.

Our study also had several limitations. First, given the inherent limitations of cross-sectional survey data, we could not test causal relationships. Second, the PRODY study survey was not designed to provide prevalence estimates stratified by levels of urbanization. Our sampling was restricted to suburban and urban areas (91% of schools were surveyed from urban areas) because it was difficult to access the population in rural areas. Therefore, more efforts should be devoted to sampling the rural population in future studies.

## Conclusions

In this survey, we found that children aged 8 to 13 years in northern China and boys aged 3 to 6 years in western China had the highest prevalence estimates of obesity. Children in northern, western, and central China had high prevalence estimates of overweight. Boys had higher prevalence estimates of obesity than girls only in eastern and northern China.

## Notes

Supplement. **eFigure 1.** Sampling Procedure **eFigure 2.** Recruitment Flow Chart, Prevalence and Risk Factors for Obesity and Diabetes in Youth (PRODY) Study, 2017-2019 **eTable 1.** Levels of Urbanization and Economic Development of Provinces, Autonomous Regions, and Municipalities in China (2016) **eTable 2.** Body Mass Index (BMI) Cutoff Values for Overweight and Obesity **eTable 3.** Growth Standards for Chinese Children
