# Error in Affiliation

**DOI:** 10.1001/jamanetworkopen.2021.38362

**Published:** 2021-11-11

In the Original Investigation titled “Regional Disparities in Obesity Among a Heterogeneous Population of Chinese Children and Adolescents,”^1^ published October 26, 2021, there was an error in the affiliation of coauthor RongXiu Zheng. The correct location of the Department of Pediatrics at Tianjin Medical University General Hospital is Tianjin, China, not Heping, China. This article has been corrected.^1^
