# Supplementary material for: Regional Disparities in Obesity Among a Heterogeneous Population of Chinese Children and Adolescents
Source: JAMA Netw Open. 2021 Oct 26;4(10):e2131040. doi: 10.1001/jamanetworkopen.2021.31040 (PMC8548942; doi:10.1001/jamanetworkopen.2021.31040)
Supplement: Supplement. — eFigure 1. Sampling Procedure eFigure 2. Recruitment Flow Chart, Prevalence and Risk Factors for Obesity and Diabetes in Youth (PRODY) Study, 2017-2019 eTable 1. Levels of Urbanization and Economic Development of Provinces, Autonomous Regions, and Municipalities in China (2016) eTable 2. Body Mass Index (BMI) Cutoff Values for Overweight and Obesity eTable 3. Growth Standards for Chinese Children [file jamanetwopen-e2131040-s001.pdf]

Supplementary Online Content

Zhang L, Chen J, Zhang J, et al. Regional disparities in obesity among a heterogeneous population of Chinese children and adolescents. *JAMA Netw Open.* 2021;4(10):e2131040. doi:10.1001/jamanetworkopen.2021.31040

eFigure 1. Sampling Procedure
eFigure 2. Recruitment Flow Chart, Prevalence and Risk Factors for Obesity and Diabetes in Youth (PRODY) Study, 2017-2019
eTable 1. Levels of Urbanization and Economic Development of Provinces, Autonomous Regions, and Municipalities in China (2016)
eTable 2. Body Mass Index (BMI) Cutoff Values for Overweight and Obesity
eTable 3. Growth Standards for Chinese Children

This supplementary material has been provided by the authors to give readers additional information about their work.

© 2021 Zhang L et al. *JAMA Network Open.*

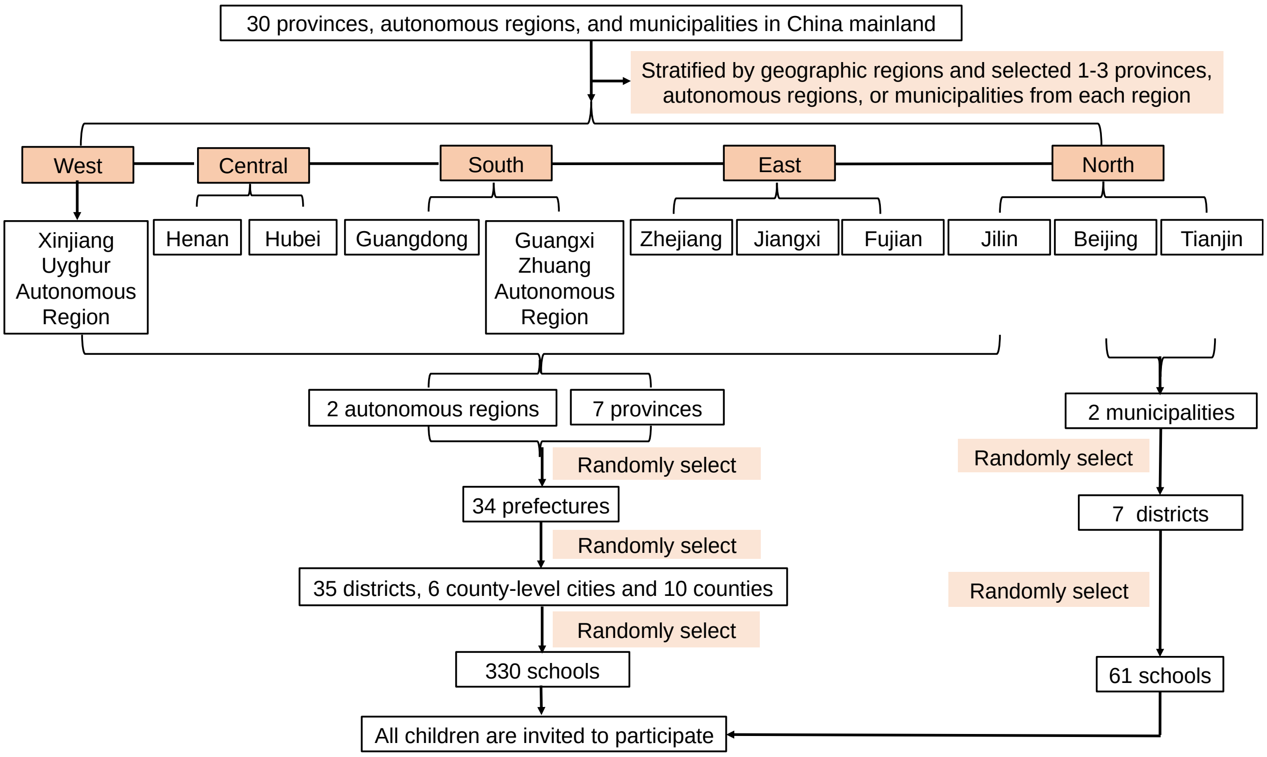

eFigure 1. Sampling Procedure

Note: We did not indicate the numbers of kindergartens, primary schools, middle schools and high schools surveyed in this figure, because it was difficult to identify the school type for some schools spanning several school types.

© 2021 Zhang L et al. *JAMA Network Open.*

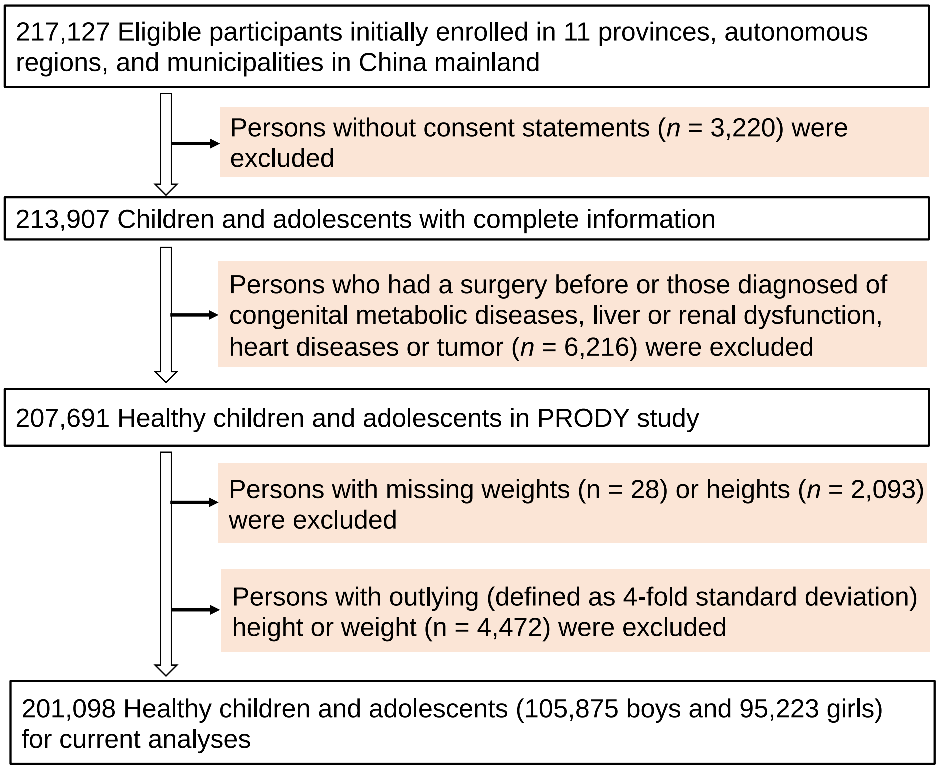

eFigure 2. Recruitment Flow Chart, Prevalence and Risk Factors for Obesity and Diabetes in Youth (PRODY) Study, 2017-2019

© 2021 Zhang L et al. *JAMA Network Open.*

eTable 1. Levels of Urbanization and Economic Development of Provinces, Autonomous Regions, and Municipalities in China (2016)

	**Provinces**	**Regional gross domestic product (100 million Yuan)**	**Urbanization ratio (%)**
North	Beijing	23014.59	86.50
	Tianjin	16538.19	82.64
	Hebei	29806.11	51.33
	Shanxi	12766.49	55.03
	Inner Mongolia Autonomous Region	17831.51	60.30
	Liaoning	28669.02	67.35
	Jinlin	14063.13	55.31
	Heilongjiang	15083.67	58.80
East	Shanghai	25123.45	87.60
	Jiangsu	70116.38	66.52
	Zhejiang	42886.49	65.80
	Anhui	22005.63	50.50
	Fujian	25979.82	62.60
	Jiangxi	16723.78	51.62
	Shandong	63002.23	57.01
Central	Henan	37002.16	46.85
	Hubei	29550.19	56.85
	Hunan	28902.21	50.89
South	Guangdong	72812.55	68.71
	Guangxi Zhuang Autonomous Region	16803.12	47.06
	Hainan	3702.76	55.12
West	Chongqing	15717.27	60.94
	Sichuan	30053.10	47.69
	Guizhou	10502.56	42.01
	Yunnan	13619.17	43.33
	Xizang	1026.39	27.74
	Shananxi	18021.86	53.92
	Gansu	6790.32	43.19
	Qinghai	2417.05	50.30
	Ningxia	2911.77	55.23
	Xinjiang Autonomous Region	9324.80	47.23

Notes: Urbanization ratio is expressed as the proportion of urban and rural populations based on the recorded household registration system in the government’s Statistical Yearbook (2016). The underlined regions are surveyed in PRODY study.

© 2021 Zhang L et al. *JAMA Network Open.*

eTable 2. Body Mass Index (BMI) Cutoff Values for Overweight and Obesity

Age	BMI cut-off value for overweight		BMI cut-off value for obesity	
	Boys	Girls	Boys	Girls
3	16.8	16.9	18.1	18.3
3.5	16.6	16.8	17.9	18.2
4	16.5	16.7	17.8	18.1
4.5	16.4	16.6	17.8	18.1
5	16.5	16.6	17.9	18.2
5.5	16.6	16.7	18.1	18.3
6	16.8	16.7	18.4	18.4
6.5	17.0	16.8	18.8	18.6
7	17.2	16.9	19.2	18.8
7.5	17.5	17.1	19.6	19.1
8	17.8	17.3	20.1	19.5
8.5	18.2	17.6	20.6	19.9
9	18.2	17.9	21.1	20.4
9.5	18.9	18.3	21.7	20.9
10	19.3	18.7	22.2	21.5
10.5	19.7	19.1	22.7	22.1
11	20.1	19.6	23.2	22.7
11.5	20.4	20.1	23.7	23.3
12	20.8	20.5	24.2	23.9
12.5	21.2	21.0	24.6	24.4
13	21.5	21.4	25.1	25.0
13.5	21.8	21.8	25.5	25.5
14	22.1	22.2	25.8	25.9
14.5	22.4	22.5	26.2	26.3
15	22.7	22.8	26.5	26.7
15.5	22.9	23.1	26.8	27.0
16	23.2	23.3	27.0	27.2
16.5	23.4	23.5	27.3	27.4
17	23.6	23.7	27.5	27.6
17.5	23.8	23.8	27.8	27.8
18	24.0	24.0	28.0	28.0

Data source: Li H, Zong XN, Ji CY, Mi J. Body mass index cut-offs for overweight and obesity in Chinese children and adolescents aged 2-18 years. *Chin J Epidemiol.* 2010;31(6):616-620.

© 2021 Zhang L et al. *JAMA Network Open.*

eTable 3. Growth Standards for Chinese Children

Age	Sex	Median height/cm	Height SD/cm	Median weight/Kg	Weight SD/Kg
3.0	Female	95.6	99.4	14.13	15.83
3.5	Female	99.4	103.3	15.16	17.01
4.0	Female	103.1	107.0	16.17	18.19
4.5	Female	106.7	110.9	17.22	19.42
5.0	Female	110.2	114.5	18.26	20.66
5.5	Female	113.5	118.0	19.33	21.98
6.0	Female	116.6	121.2	20.37	23.27
6.5	Female	119.4	124.3	21.44	24.61
7.0	Female	122.5	127.6	22.64	26.16
7.5	Female	125.6	130.8	23.93	27.83
8.0	Female	128.5	133.9	25.25	29.56
8.5	Female	131.3	136.9	26.67	31.45
9.0	Female	134.1	139.9	28.19	33.51
9.5	Female	137.0	143.1	29.87	35.82
10.0	Female	140.1	146.4	31.76	38.41
10.5	Female	143.3	149.8	33.80	41.15
11.0	Female	146.6	153.3	36.10	44.09
11.5	Female	149.7	156.3	38.40	46.87
12.0	Female	152.4	158.8	40.77	49.54
12.5	Female	154.6	160.8	42.89	51.75
13.0	Female	156.3	162.3	44.79	53.55
13.5	Female	157.6	163.4	46.42	54.99
14.0	Female	158.6	164.3	47.83	56.16
14.5	Female	159.4	164.9	48.97	57.06
15.0	Female	159.8	165.3	49.82	57.72
15.5	Female	160.1	165.6	50.45	58.19
16.0	Female	160.1	165.5	50.81	58.45
16.5	Female	160.2	165.6	51.07	58.64
17.0	Female	160.3	165.7	51.20	58.73
17.5	Female	160.3	165.7	51.20	58.73
18.0	Female	160.6	165.9	51.41	58.88
18.5	Female	160.6	165.9	51.41	58.88
3.0	Male	96.8	100.7	14.65	16.39
3.5	Male	100.6	104.5	15.63	17.50
4.0	Male	104.1	108.2	16.64	18.67
4.5	Male	107.7	111.9	17.75	19.98
5.0	Male	111.3	115.7	18.98	21.46
5.5	Male	114.7	119.2	20.18	22.94

© 2021 Zhang L et al. *JAMA Network Open.*

Age	Sex	Median height/cm	Height SD/cm	Median weight/Kg	Weight SD/Kg
6.0	Male	117.7	122.4	21.26	24.32
6.5	Male	120.7	125.6	22.45	25.89
7.0	Male	124.0	129.1	24.06	28.05
7.5	Male	127.1	132.4	25.72	30.33
8.0	Male	130.0	135.5	27.33	32.57
8.5	Male	132.7	138.4	28.91	34.78
9.0	Male	135.4	141.2	30.46	36.92
9.5	Male	137.9	144.0	32.09	39.12
10.0	Male	140.2	146.4	33.74	41.31
10.5	Male	142.6	149.1	35.58	43.69
11.0	Male	145.3	152.1	37.69	46.33
11.5	Male	148.4	155.4	39.98	49.19
12.0	Male	151.9	159.4	42.49	52.31
12.5	Male	155.6	163.3	45.13	55.54
13.0	Male	159.5	167.3	48.08	59.04
13.5	Male	163.0	170.5	50.85	62.16
14.0	Male	165.9	173.1	53.37	64.84
14.5	Male	168.2	175.0	55.43	66.86
15.0	Male	169.8	176.3	57.08	68.35
15.5	Male	171.0	177.3	58.39	69.44
16.0	Male	171.6	177.8	59.35	70.20
16.5	Male	172.1	178.2	60.12	70.79
17.0	Male	172.3	178.4	60.68	71.20
18.0	Male	172.7	178.7	61.40	71.73

Data source: Department of Maternal and Child Health and Community Health, Ministry of Health, People's Republic of China; Coordinating Study Group of Nine Cities on the Physical Growth and Development of Children; Capital institute of pediatrics. Growth standards and growth charts for Chinese children. Second Military Medical University Press; 2009.

© 2021 Zhang L et al. *JAMA Network Open.*
